# A comparison of the effects of fire needle and routine acupuncture for myofascitis: A protocol for systematic review and meta-analysis

**DOI:** 10.1097/MD.0000000000025473

**Published:** 2021-06-11

**Authors:** Wei Xiong, Ling Cheng, Zhiying Zhong, Xinju Hou, Manhua Zhu, Xingchen Zhou, Siyuan Zhu, Jun Chen

**Affiliations:** a Nanchang Hongdu Hospital of Traditional Chinese Medicine; b Jiangxi University of Traditional Chinese Medicine, Nanchang, PR China.

**Keywords:** fire needle, meta-analysis, myofascitis, protocol, routine acupuncture, systematic review

## Abstract

**Background:**

Myofascitis is a common disease in clinic. The main cause of the disease is aseptic inflammation of local muscles and connective tissues such as myofascial, which can be manifested as paralysis, distension, and other discomfort, local muscle stiffness, spasm or palpable strain-like nodules. Chinese medicine ascribes it to “bi disease” and “Arthralgia disease,” while Western medicine believes that the disease is mainly due to local muscle and fascia edema and exudation caused by trauma or long-term strain, forcing nerves to jam and producing pain and other abnormal feelings. Although the disease is not life-threatening, the pain and distension caused by local inflammatory stimuli can affect the patient's daily life and sleep quality. The purpose of this systematic review is to evaluate the efficacy of fire needle vs routine acupuncture in the treatment of myofascitis.

**Methods:**

Randomized controlled trials (RCTS) of fire needle vs routine acupuncture for myofascial inflammation will be comprehensively searched from inception to September 2020 on PubMed, Embase, Cochrane Library, China Biomedical Literature (CBM), China National Knowledge Infrastructure (CNKI), Chongqing VIP (CQVIP), and Wanfang. Additionally, RCT registered sites, including http://www.ClinicalTrials.gov and http://www.chictr.org.cn, also will be the search. Visual analogue scale (VAS) was used to score the pain before and after treatment. The primary outcome will be to compare the difference in pain scores between the 2 interventions. Two independent authors filtered the literature in the above database, extracted the data, and cross-checked it.

**Results:**

This study will offer a reasonable comprehensive evidence for the treatment of myofascitis with fire needle.

**Conclusion:**

The conclusion of this study will provide evidence to judge the effectiveness of fire needle on myofascitis.

**Registration number:**

INPLASY202080034.

## 1 Introduction

### 1.1 Description of the condition

Also known as myofascial pain symptoms (MPS), myofascitis is a common soft-tissue rheumatism characterized by chronic muscle pain with one or more trigger points.^[1,2]^ It is mainly manifested as chronic pain caused by aseptic inflammation,^[3]^ the most common and specific symptoms are referral and recurrent musculoskeletal pain, which is commonly seen clinically in cervical and shoulder, lumbar and back myofascitis. The International Association for the Study of Pain points out that myofascial pain syndrome is a common source of musculoskeletal pain.

To understand how trigger points are associated with the sensory and motor symptoms of MPS, we investigated etiological factors. Myofascial trigger points can be caused by acute or repetitive muscle injury or overloading, physical disease, joint injury, poor posture, spinal and disc lesions, and systemic diseases such as fibromyalgia.^[4,5]^ Several studies have suggested that acetylcholine leakage may play an important role in the MPS process. Acetylcholine leakage causes damage to the sarcoplasmic reticulum, leading to the release of large amounts of calcium, which in turn leads to secondary myoconstriction and cell membrane damage.^[6,7]^ Eventually, as the muscles continue to contract and remain excited, ischemia, hypoxia, and a large amount of calcium are released, and the cycle begins. Some studies have shown that inflammatory cytokines are associated with MPS.^[8,9]^ Inflammatory factor substance P, interleukin-1beta, tumor necrosis factor, 5-hydroxytryptamine are relatively high in the muscle area of trigger point,^[10]^ and participate in all processes related to the mechanisms of pain.^[11]^ A recent study by Xie et al showed that individuals with chronic pain associated with myofascial trigger showed microstructural changes in gray matter in brain regions associated with the limbic system and pain stroma.^[12]^

Interventions for MPS aim to relieve pain and improve mobility and function. First-line options for MPS are drug therapy and noninvasive therapy.^[13]^ The most commonly used pain-relieving drug for most pain syndromes (including MPS) is nonsteroidal anti-inflammatory drugs (NSAIDs), which have mild side effects on the digestive system. Although oral nonsteroidal anti-inflammatory drugs (NSaids) are widely used in clinical practice, there are no randomized controlled trials (RCTS) specifically evaluating the treatment of MPS. Therefore, there is no strong evidence for the anti-inflammatory effects of MPS.^[14]^ However, several studies have shown strong evidence for the use of NSaids in the treatment of low back pain.^[15,16]^ Since MPS resembles the symptoms of low back pain, it is reasonable to assume that nonsteroidal anti-inflammatory drugs (NSaids) have similar efficacy in treating both pain syndromes. In summary, the potential adverse effects of long-term use of NSaids on the gastrointestinal tract, kidneys, and antiplatelets are obvious disadvantages that should be treated with caution by both doctors and patients.^[17]^ Muscle relaxants are commonly used to reduce muscle cramps. One study demonstrated that tizanidine not only significantly reduced pain intensity and dysfunction, but also improved sleep duration and quality.^[18]^ As tizanidine is effective in treating low back pain, some researchers have suggested that tizanidine should be used as the primary treatment for MPS.^[18]^ Anticonvulsants like Pregabalin provide analgesia, antianxiety, and anticonvulsants, and are effective in reducing the release of neurochemicals such as glutamate, norepinephrine, and substance P.^[19]^ Two RCTS assessed the efficacy of Tizanidine in the treatment of acute low back pain, and the results showed that tizanidine was more effective in reducing pain than placebo.^[20,21]^ It appears that Tezanidine may have a similar therapeutic effect on MPS, but strong evidence is lacking. Cyclobenzaprine is one of the most widely studied anticonvulsant drugs, effective in relieving pain associated with skeletal muscle spasm and acute musculoskeletal disease.^[22]^ Two randomized controlled trials demonstrated the efficacy of cyclobenzaprine in patients with MPS.^[23,24]^ Another RCT study evaluating cyclobenzalin did not find its effectiveness.^[25]^ Therefore, there is currently insufficient evidence to support cyclobenzaprine as a treatment for MPS. There are many other drugs such as tranquilizers, hypnotics, and antidepressants available for treatment, but the main and preferred treatment is still no consensus.

As a safe and effective alternative therapy, acupuncture and moxibustion is gradually accepted by all countries in the world. Previous studies have shown that fire needle has a positive effect on the pain of myofascial inflammation. But no systematic review or research scheme has been published so far. We therefore had the opportunity to evaluate this issue and present a systematic review based on the most comprehensive and up-to-date resources to determine the effectiveness of fire in patients with myofascitis.

### 1.2 Description of the intervention

Acupuncture is an important part of traditional Chinese medicine (TCM), in which fine needles are inserted into specific parts of the body. It was developed and practiced from the Shang Dynasty in ancient China (1600 BC 1100). Acupuncture is based on the basic theory of TCM, which holds that maintaining a balance of energy means good health, and that the so-called Qi constantly flows through the body's channels and collaterals. The therapeutic effect of acupuncture is to correct the imbalance of qi and blood flow by inserting needles at specific points on the meridians.^[26]^ Acupuncture has been widely used to treat a variety of diseases in China for more than 2000 years, proving its effectiveness. Due to the good effect of acupuncture on analgesia, it has gradually attracted worldwide attention. In 1980, acupuncture was recommended by the World Health Organization as an alternative treatment for 43 different diseases. A survey has found that 8 million locals in the United States have been treated for acupuncture, most commonly for lower back pain.^[27,28]^ Recent studies have shown that acupuncture not only reduces pain immediately, but also promotes the recovery of systemic function. The fire needle is developed on the basis of acupuncture and moxibustion. As a valuable treasure of traditional Chinese medicine, it has attracted wide attention all over the world. It has been widely used in clinical treatment and achieved remarkable results. In facial spasm, periarthritis of shoulder and other neurovascular conflicts, the advantage of fire needle therapy has been confirmed. A number of studies have shown that fire needles can be beneficial in the treatment of myofascitis. However, the efficacy of fire needles in the treatment of myofascitis remains unclear due to the lack of available evidence that is still fully assessed. Therefore, our ultimate goal is to systematically and comprehensively evaluate all randomized controlled trials (RCTS) on fire needle therapy for myofascial inflammation, providing reliable evidence for clinical treatment of myofascial inflammation.

## 2 Methods

### 2.1 Registration

This review will be based on the preferred reporting items for systematic review and meta-analysis protocol.^[29]^ It is registered in the INPLASY (registration number, INPLASY202080034; https://inplasy.com/inplasy-2020-8-0034/).

### 2.2 Inclusion criteria for study selection

#### 2.2.1 Types of study

In order to evaluate the therapeutic effect of fire needle on MPS, this paper only reviews the RCT of fire needle and control group, such as ordinary acupuncture, drug therapy, placebo treatment, etc. All unrestricted RCTS will be included, and nonrandomized controlled trials, review reports, case reports, and animal studies will be excluded. In addition, the language limit is Chinese and English.

#### 2.2.2 Types of participants

Patients meeting the diagnostic criteria for MPS regardless of sex, age, race, education, and economic status.^[30,31]^

#### 2.2.3 Types of intervention

The study used fire needles or in combination with other therapies, without limiting the duration and dose of treatment.

#### 2.2.4 Types of outcome measures

The main result of this review was that body function was preferentially extracted from the visual simulation scale. Secondary results including the cervical spondylosis, the Japanese orthopedic association scoring, have dysfunction index, the American orthopedic foot and Ankle Society - Ankle Hindfoot scale, the ability of the foot and Ankle, Cumberland Ankle instability tools, Pittsburgh sleep quality index, self-evaluation anxiety scale, self - rating scale, Pittsburgh sleep quality index, the recurrence rate, period, adverse events.^[31,32]^ The system review will be conducted independently.

#### 2.2.5 Exclusion criteria

- Participants with the unclear diagnosis;
- Studies that did not use fire needle therapy as primary treatment in the intervention group;
- Data that cannot be extracted;
- Duplicated data;
- The studies where full text is unavailable.

### 2.3 Data sources

Our systematic review will electronically retrieve all RCTS for fire needle therapy of MPS prior to December 2020, no matter the publication status or language. The databases include PubMed, Embase, the Cochrane Library, CNKI, Chinese VIP information, Wanfang Database and CBM. Other sources of information, such as bibliographies of identified publications and minutes of meetings, will also be searched, and statistical analyses will be conducted using the Review Manager V.5.4.

### 2.4 Search strategy

The search will follow PRISMA's guidelines. The following key search terms will be used: (“myofascitis” OR “myofascial pain” OR “myofascial syndrome” OR “cervicoshoulder myofascitis” OR “lumbar myofascitis”) AND (“fire needle” OR “fire acupuncture”) AND (“randomized”). The search strategy will adapt to different database requirements. The search strategy in PubMed is shown in Table 1.

**Table 1:** Search strategy (PubMed).

Order	Strategy
#1	Search “myofascitis” [Mesh] OR “Myofascial pain” [Mesh] OR “Myofascial syndrome” [Mesh] Sort by: publication date
#2	Search (((((myofascitis [Title/Abstract]) OR Myofascial pain [Title/Abstract]) OR Myofascial syndrome [Title/Abstract]) OR cervicoshoulder myofascitis [Title/Abstract]) OR lumbar myofascitis [Title/Abstract]) Sort by: publication date
#3	#1 OR #2
#4	Search (((((((randomized controlled trial [Publication Type]) OR controlled clinical trial [Publication Type]) OR randomized [Title/Abstract]) OR drug therapy [Mesh Subheading]) OR placebo [Title/Abstract]) OR randomly [Title/Abstract]) OR trial [Title/Abstract]) OR groups [Title/Abstract] Sort by: Publication Date
#5	Search “fire needle” [Mesh]) OR “fire acupuncture” [Mesh] OR “acupuncture” [Mesh T] Sort by: publication date
#6	#4 AND #5
#7	#3 AND #6

### 2.5 Data collection and analysis

#### 2.5.1 Selection of studies

We will select the RCTs that compare the efficacy of fire needles in the treatment of myofascitis. Clauses that conform to one of the following provisions will not be included:

1. the duplicates,
2. the participants did not meet the diagnosis criteria of myofascitis or were not known,
3. not RCT studies,
4. participants in the study did not receive a combination of fire needle and conventional therapy as the primary intervention,
5. the intervention contains any other traditional Chinese medicine (TCM) therapy,
6. incomplete data required.

**Figure 1:**
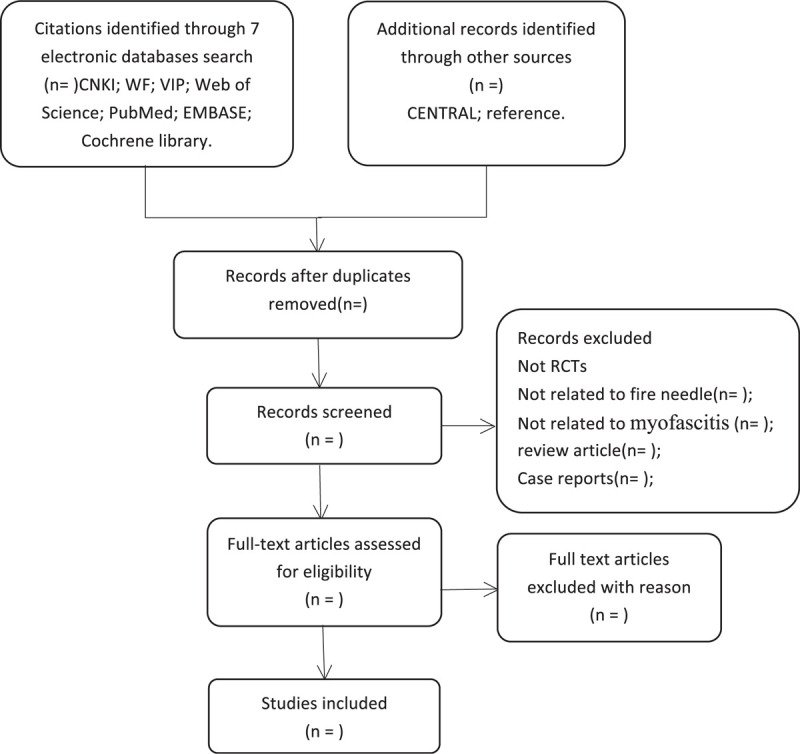
Flowchart of literature selection.

It will be up to the authors to assess whether these studies meet the requirements. If there are any objections to any of the terms included, we will discuss them together and resolve them. The specific process of study selection will be shown in the flow chart of preferred reporting items in the Systematic Review and Meta-analysis (PRISMA) (Fig. 1).

#### 2.5.2 Data extraction and management

The data will be extracted by 2 researchers using a predefined data collection form. The extracted data shall include, but not be limited to, the following items: title, first author, time of publication, sample size, age and gender of participants, outcomes, and adverse events. For papers with incomplete or unclear information, we will try to obtain the missing information from the corresponding author by email or telephone. Any differences that arise will be discussed and resolved by 2 of the authors, and any further differences will be arbitrated by a third author.

### 2.6 Assessment of risk of bias and reporting of study quality

The authors (GHT and JC) will use the Cochrane Collaboration Risk assessment tool to assess the risk of bias for all included studies. We will assess the risk of bias in sequence generation, allocation sequence concealment, blindness of participants and staff, outcome evaluators, incomplete outcome data, selective outcome reporting, and other sources of bias. This review USES L, U, and H as the key to evaluation, where L(low) means low risk of bias, U(unclear) means uncertain risk of bias, and H(high) means high risk of bias. In the event of inconsistent results, the final decision is made by the third author (LBL). This study provides a tabular summary of the information contained in the risk assessment for bias and critically discusses its results and implications. If the information is not fully understood, we will try to contact the author. For duplicate publications, we select only the earliest published text.

#### 2.6.1 Measures of treatment effect

RevManV.5.4 was used for data analysis and quantitative data synthesis. For continuous data, if no heterogeneity exists, we will use mean difference or standard mean deviation to measure the treatment effect of 95% confidence interval (CI). If significant heterogeneity is found, random-effects models will be used. For dichotomous data, we will use 95% CI risk ratio for analysis.

#### 2.6.2 Unit of analysis issues

Data from parallel design studies were included for meta-analysis. Only phase I data will be included in randomized cross-over trials. In these trials, participants were randomly divided into 2 intervention groups, and each outcome of each participant was collected and analyzed.

#### 2.6.3 Management of missing data

If missing or incomplete data affects the main results, we will attempt to contact the corresponding author to obtain the missing data. If it is still not available, the experimental data is excluded from the analysis.

### 2.7 Assessment of heterogeneity

We will use RevMan to assess efficacy and publication bias (version 5.4, Nordic Cochrane Center, Copenhagen, Denmark). The forest map is used to show the relative strength of the effect, and the funnel plot is used to show the deviation. If significant differences are detected, a random effects model is used.

#### 2.7.1 Assessment of reporting biases

We will use funnel plots to detect reported deviations. If more than 10 trials are included, funnel plots will be used to assess reported bias. If the funnel plot is found to be asymmetric, the Egger method is used to analyze the reason. We will include all eligible tests regardless of the quality of the method.

#### 2.7.2 Data synthesis

We will use RevMan for all statistical analysis. If considerable heterogeneity is observed, joint effect estimates are analyzed using a 95% CI stochastic effects model. If necessary, each subgroup will be analyzed carefully.

#### 2.7.3 Subgroup analysis

There is no pregrouping plan. Subgroup analysis was performed based on control interventions and different outcomes.

#### 2.7.4 Sensitivity analysis

Sensitivity analysis will be performed based on sample size, heterogeneity quality, and statistical models (random or fixed effects models).

#### 2.7.5 Grading the quality of evidence

The quality of evidence for all results will be judged by the grading of the methodology of the recommended assessment, formulation and assessment Working Group. Bias risk, consistency, directness, accuracy, and publication bias were evaluated. High, medium, low, or very low represent 4 ratings.^[33–35]^

## 3 Discussion

Pain is one of the common reasons people go to the hospital. Most patients with musculoskeletal pain disorder are diagnosed with myofascitis, which is a major cause of pain syndrome and affects a large number of patients around the world. Despite the variety of drugs used to treat MPS, the treatment results are not satisfactory to the majority of patients. Supplementary treatment should also be considered to compensate for the deficiency. The evaluation of this system review will be divided into 4 parts: identification, literature inclusion, data extraction, and data comprehensive analysis. Based on the Cochrane method, this study searched and screened the major electronic literature databases of evidence-based medicine through the analysis of clinical RCT evidence at home and abroad, so as to provide more convincing evidence for clinicians to make decisions and better guide clinical treatment.

Although there have been some SRs and meta-analyses on the treatment of myofascitis, the efficacy of fire acupuncture in the treatment of myofascitis has not been reviewed. At present, there is no relevant literature to evaluate the methods and report quality of these studies. Although the potential low quality of the original RCT may affect the reliability of this systematic review, it is meaningful to conduct this study. This systematic study will integrate all RCT studies on the treatment of myofascial inflammation with fire needle.

## Author contributions

**Conceptualization:** Zhiying Zhong, Siyuan Zhu.

**Data curation:** Wei Xiong, Zhiying Zhong, Ling Cheng, Xinju Hou, Manhua Zhu, Xingchen Zhou, Siyuan Zhu, Jun Chen.

**Formal analysis:** Xinju Hou, Manhua Zhu, Siyuan Zhu, Jun Chen.

**Investigation:** Wei Xiong, Zhiying Zhong, Ling Cheng.

**Methodology:** Xinju Hou, Manhua Zhu, Siyuan Zhu, Jun Chen.

**Software:** Siyuan Zhu, Jun Chen.

**Supervision:** Wei Xiong, Ling Cheng, Xinju Hou, Manhua Zhu, Xingchen Zhou.

**Writing – original draft:** Wei Xiong, Zhiying Zhong, Ling Cheng, Xinju Hou.

**Writing – review & editing:** Wei Xiong, Ling Cheng, Xinju Hou, Manhua Zhu.

## References

[1] Kehler T. Myofascial pain syndrome. Reumatizam 2013;60:81–3. 24980001

[2] Akamatsu FE Ayres BR Saleh SO. Trigger points: an anatomical substratum. BioMed Res Int 2015;2015:05. 10.1155/2015/623287 PMC4355109 25811029

[3] Haviv Y Rettman A Aframjan D. Myofascial pain: an open study on the pharmaeotherapeutic pesponse to stepped treatment with tricyclic antidepressants and gabapentin. J Oral Facial Pain Headache 2015;29:144–51. 25905532 10.11607/ofph.1408

[4] Morihisa R Eskew J McNamara A. Dry needling in subjects with muscular trigger points in the lower quarter: a systematic review. Int J Sports Phys Ther 2016;11:01–4. PMC4739038 26900495

[5] Cojocaru MC Cojocaru IM Voiculescu VM. Trigger points-ultrasound and thermal findings. J Med Life 2015;8:315–8. 26351532 PMC4556911

[6] Zhuang X Tan S Huang Q. Understanding of myofascial trigger points. Chin Med J 2014;127:4271–7. 25533832

[7] Bron C Dommerholt JD. Etiology of myofascial trigger points. Curr Pain Headache Rep 2012;16:439–44. 22836591 10.1007/s11916-012-0289-4 PMC3440564

[8] Bruckle W Suckfull M Fleckenstein W. Tissue pO2 measurement in taut back musculature (m. erector spinae). Z Rheumatol 1990;49:208–16. 2146822

[9] Bengtsson A Henriksson KG Larsson J. Reduced high-energy phosphate levels in painful muscle in patients with primary fibromyalgia. Arthritis Rheum 1986;29:817–21. 3741498 10.1002/art.1780290701

[10] Shah JP Gilliams EA. Uncovering the biochemical milieu of myofascial trigger points using in vivo microdialysis: an application of muscle pain concepts to myofascial pain syndrome. J Bodyw Mov Ther 2008;12:371–84. 19083696 10.1016/j.jbmt.2008.06.006

[11] Kale S Biermann S Edwards C. Three-dimensional cellular development is essential for ex vivo formation of human bone. Nat Biotechnol 2000;18:954–8. 10973215 10.1038/79439

[12] Xie P Qin B Song G. Microstructural abnormalities were found in brain gray matter from patients with chronic myofascial pain [published correction appears in Front Neuroanat. 2017 Jul 21; 11 62]. Front Neuroanat 2016;10:122. 28066193 10.3389/fnana.2016.00122 PMC5167736

[13] Desai MJ Saini V Saini S. Myofascial pain syndrome: a treatment review. Pain Ther 2013;2:21–36. 25135034 10.1007/s40122-013-0006-y PMC4107879

[14] Fomby E Mellion M. Identifying and treating myofascial pain syndrome. Phys Sport Med 1997;25:67–75. 10.3810/psm.1997.02.1674 20086886

[15] Lacey P Dodd G Shannon D. A double blind, placebo-controlled study of piroxicam in the management of acute musculoskeletal disorders. Eur J Rheumatol Inflamm 1984;7:95–104. 6443759

[16] van Tulder M Koes B Bouter L. Conservative treatment of acute and chronic nonspecific low back pain: a systematic review of randomized controlled trials of the most common interventions. Spine (Phila Pa 1976) 1997;22:2128–56. 9322325 10.1097/00007632-199709150-00012

[17] Amlie E Weber H Holme I. Treatment of acute low-back pain with piroxicam: results of a double-blind placebo-controlled trial. Spine (Phila Pa 1976) 1987;12:473–6. 2957801 10.1097/00007632-198706000-00010

[18] Malanga G Gwynn M Smith R. Tizanidine is effective in the treatment of myofascial pain syndrome. Pain Physician 2002;5:422–32. 16886022

[19] Crofford L Rowbotham M Mease P. Pregabalin for the treatment of fibromyalgia syndrome. Arthritis Rheum 2005;52:1264–73. 15818684 10.1002/art.20983

[20] Berry H Hutchinson DR. Tizanidine and ibuprofen in acute low-back pain: results of a double-blind multicentre study in general practice. J Int Med Res 1988;16:83–91. 2967781 10.1177/030006058801600202

[21] Berry H Hutchinson DR. A multicentre placebo-controlled study in general practice to evaluate the efficacy and safety of tizanidine in acute low-back pain. J Int Med Res 1988;16:75–82. 2967780 10.1177/030006058801600201

[22] Leite F Atallah A El Dib R. Cyclobenzaprine for the treatment of myofascial pain in adults. Cochrane Database Syst Rev 2009. CD006830. 19588406 10.1002/14651858.CD006830.pub3 PMC6481902

[23] Furtado RNV Carazzato S Farias CA. Myofascial syndrome: comparison between infiltration of trigger points treatment and oral medication (cyclobenzaprine). Acta Fisiátrica 2002;9:117–26.

[24] Turturro M Frater C D’Amico F. Cyclobenzaprine with ibuprofen versus ibuprofen alone in acute myofascial strain: a randomized, double blind clinical trial. Ann Emerg Med 2003;41:818–26. 12764337 10.1067/mem.2003.188

[25] Herman CR Schiffman EL Look JO. The effectiveness of adding pharmacologic treatment with clonazepam or cyclobenzaprine to patient education and self-care for the treatment of jaw pain upon awakening: a randomized clinical trial. J Orofac Pain 2002;16:64–70. 11889661

[26] Amezaga Urruela M Suarez-Almazor ME. Acupuncture in the treatment of rheumatic diseases. Curr Rheumatol Rep 2012;14:589–97. 23055010 10.1007/s11926-012-0295-x PMC3691014

[27] Burke A Upchurch DM Dye C. Acupuncture use in the United States: findings from the National Health Interview Survey. J Altern Complement Med 2006;12:639–48. 16970534 10.1089/acm.2006.12.639

[28] World Health Organization. Report of the working group on auricular acupuncture nomenclature. XXXX 1991. 25.

[29] Larissa S David M Mike C. Preferred reporting items for systematic review and meta-analysis protocols (PRISMA-P) 2015: elaboration and explanation. BMJ 2015;349:7647–17647.

[30] Gerwin RD Shannon S Hong CZ. Interrater reliability in myofascial trigger point examination. Pain 1997;69:65–73. 9060014 10.1016/s0304-3959(96)03248-4

[31] Travell JG Simons DG. Myofascial Pain and Dysfunction: The Trigger Point Manual. Vol 1. 2 Edition edn. Baltimore, MD: Williams and Wilkins; 1998.

[32] Yeh CH Suen LK Shen J. Changes in sleep with auricular point acupressure for chronic low back pain. Behav Sleep Med 2016;14:279–94. 26244591 10.1080/15402002.2014.981820

[33] Ünal Ö Akyol Y Tander B. The relationship of illness perceptions with demographic features, pain severity, functional capacity, disability, depression, and quality of life in patients with chronic low back pain. Turkish J Phys Med Rehabil 2019;65:301–8. 10.5606/tftrd.2019.3248 PMC6935732 31893266

[34] Alaca N Kaba H Atalay A. Associations between the severity of disability level and fear of movement and pain beliefs in patients with chronic low back pain. J Back Musculoskelet Rehabil 2019;33:785–91. 10.3233/BMR-171039. 10.3233/BMR-171039 31868657

[35] Rickards L. The effectiveness of non-invasive treatments for active myofascial trigger point pain: a systematic review of the literature. Int J Osteopath Med 2009;12:42–3.

